# Primary School Teachers’ Adaptations for Struggling Writers: Survey Study of Grade 1 to 6 Teachers in Australia

**DOI:** 10.1177/00222194231211946

**Published:** 2023-11-13

**Authors:** Anabela Malpique, Deborah Pino-Pasternak, Debora Valcan, Mustafa Asil

**Affiliations:** 1 Edith Cowan University, Perth, Western Australia, Australia; 2 Universidade de Lisboa, Portugal; 3 University of Canberra, Australian Capital Territory, Australia; 4 Murdoch University, Western Australia, Australia; 5 Bond University, Robina, Queensland, Australia

**Keywords:** writing, adaptations, struggling writers, primary education

## Abstract

Two hundred ninety-eight primary teachers (88% female) from across all Australian states and territories reported on the frequency with which they implemented instructional adaptations for struggling writers in their classrooms. They also rated their preparation and self-efficacy for teaching writing. The majority of participating teachers indicated they provided additional instruction on spelling, capitalization and punctuation, and sentence construction at least once a week or more often. Teachers further reported implementing additional minilessons and reteaching strategies and skills, as well as extra instruction on grammar, handwriting, text structure, revising, and planning on a monthly basis or more often. The majority of teachers reported never or only once a year using adaptations to support digital writing. The frequency with which teachers provided extra instruction on spelling, handwriting, text structure, revising, and computer use differed by grade. Only teachers’ perceived efficacy to teach writing made a unique and statistically significant contribution to predicting the use of instructional adaptations for writing and adaptations to support digital writing after controlling for teacher and classroom variables.

Developing proficiency in writing across schooling is critical for students because it allows them to acquire knowledge in different learning areas, communicate what they learn across subjects, and develop critical thinking skills that will be invaluable when entering the workplace (United Nations Educational, Scientific and Cultural Organization [UNESCO], 2019). From different educational contexts across the world, however, reports show that many students struggle to learn how to write and finish compulsory education without adequate writing proficiency (Graham, 2019). For example, findings from the latest national assessment of writing in the United States showed that two of every three students wrote below grade-level standards (National Center for Educational Statistics, 2012). In Australia, where the current study took place, results from the National Assessment Program Literacy and Numeracy (NAPLAN) also paint a worrisome picture of students’ writing performance, with a significant decline in writing proficiency between 2011 and 2018 (Australian Curriculum, Assessment and Reporting Authority [ACARA], 2021b; Thomas, 2020). A recent longitudinal analysis of NAPLAN writing results from 2011 to 2021 (Australian Education Research Organisation [AERO], 2022) found a growing performance gap between low and high-achieving students in writing, with findings showing a significant decline in the percentage of Grades 5, 7, and 9 students who achieved the highest scores from 2011 to 2018. These results reinforce the need to provide additional support to struggling writers as early as possible because without tailored support the performance gap in writing is expected to increase as students get older (AERO, 2022).

Achievement benchmarks for writing acquisition and development are set at a national level in Australia via the Australian Curriculum, with states and territories being responsible for implementing it as is or adapting it, should they desire (ACARA, 2021b). Achievement standards set under the English subject strand include the development of foundational writing skills (e.g., handwriting, typing, and spelling) and process writing skills (e.g., planning, organizing, and editing texts). In addition, literacy is presented as a general capability under the Australian curriculum (ACARA, n.d.), and all primary and secondary teachers are expected to support the development of literacy skills, including writing. While specific instructional practices for writing are not mandated at the national and state levels, current educational policies and curriculum changes emphasize the need to develop teaching practices to cater for students’ diversity through personalized learning (ACARA, 2020). National curriculum efforts to promote an education for all pedagogy include the provision of teaching resources illustrating personalized learning and adjustments across learning areas, including in literacy (ACARA, 2021a; for a review, see Price & Slee, 2021). Providing differentiated instruction and enacting pedagogies that respond to student diversity is paramount in the Australian context. According to the most recent Teaching and Learning International Survey (TALIS) results (Organisation for Economic Cooperation and Development [OECD], 2019), secondary schools in Australia (Grades 7–10) are more diverse than the average across the OECD. Figures from the Australian Bureau of Statistics (2019) indicate that 10% of primary and secondary students in Australia have an identified disability, with 89% of these students attending mainstream schools. In terms of writing development, it is acknowledged that beyond students with identified disabilities there is a “silent majority [of students] who lack writing proficiency but do not receive additional help” (López-Escribano et al., 2022, p. 3).

Catering for the abovementioned diversity is critical when considering the complexity of writing. Theoretically, cognitive models of writing (Berninger & Winn, 2006; Hayes, 1996) unanimously present proficient writing as a complex process involving the acquisition and development of foundational writing skills, such as transcription skills (e.g., handwriting, typing, and spelling), as well as process writing skills, such as strategies for planning and revising texts. In a recent comprehensive model merging cognitive and sociocultural models of writing acquisition and development, Graham (2018) argues in his Writer(s)-within-Community (WWC) model that “writing is simultaneously shaped by the community in which it takes place and the cognitive capabilities and resources of community members who create it” (p. 271), reinforcing the need to consider micro- (students-level variables), meso- (contextual-level factors, including school and home variables), and macro-level factors (historical, cultural, and education-policy level variables) affecting effective writing development. The study reported here examined teachers’ adaptations for struggling writers in Australian primary classrooms (Grades 1–6; 6- to 12-year-olds) and the role of teachers’ preparation and self-efficacy beliefs in explaining these instructional adaptations, hence addressing meso- and macro-level influences on students’ writing development.

## Instructional Adaptations for Struggling Writers

Despite the critical role of implementing instructional adaptations for students experiencing difficulties in learning to write (Graham et al., 2003; Troia & Graham, 2017), research investigating specific adaptations that teachers typically implement for struggling writers in primary classrooms is scarce, with the majority of studies being conducted in the United States (Graham, 2019). In two national studies, Graham and colleagues (2003, 2016) surveyed Grades 1 to 3 teachers about the types and frequency of adaptations that they made for struggling writers in their classrooms. In both studies, findings suggested that the majority of teachers made little or no adaptations for struggling writers in early primary. In their national sample of 153 primary teachers, Graham and colleagues (2003) found that teachers placed a greater emphasis on teaching basic writing skills to struggling writers, such as spelling and grammar, while little focus was placed on using computers to support writing. In a subsequent survey of 125 teachers, Graham and colleagues (2016) found that Grades 1 to 3 teachers reported placing more focus on providing additional encouragement for writing and time to complete writing assignments, with both adaptations reportedly made daily or more often. Aligned with the previous 2003 survey, instruction via keyboard and using technology were the least two reported adaptations, with most teachers reporting never implementing such adaptations to support struggling writers. In another U.S. national survey examining instructional adaptations for struggling writers in a sample of 103 teachers (Grades 4–6), Gilbert and Graham (2010) found that teachers reportedly did not frequently make additional adaptations. The most frequently reported adaptations (occurring weekly or more often) were providing encouragement for writing, followed by additional time to practice writing skills and complete writing assignments. Extra writing instruction via technology was, once again, the least frequently reported adaptation. Finally, in a study investigating instructional adaptations for students with disabilities (SWDs; Troia & Graham, 2017), a sample of 141 teachers (Grades 3–8) reported using only one adaptation on a daily basis to support SWDs, namely, allowing extra time for completion of writing assignments. Interestingly, unlike findings from previous U.S. studies (Gilbert & Graham, 2010; Graham et al., 2003, 2016), about half of participating teachers reported that they allowed SWDs to use computers to compose texts at least weekly.

National surveys investigating adaptations for struggling writers outside of the U.S. context are limited to only two studies in upper-primary and middle-school contexts. Veiga Simão et al. (2016) asked Portuguese (*n* = 195) and Brazilian (*n* = 99) middle school teachers (Grades 5–9) to report on the adaptations that they typically implemented to support struggling writers in their classrooms. Contrary to findings from the U.S. context, teachers in both countries reported making adaptations at least weekly or more often, placing more recurrent focus on additional time to complete writing tasks and reteaching writing skills previously taught. In a more recent study, Graham et al. (2022) examined the instructional adaptations that a sample of 254 teachers (Grades 4–6) implemented in Chile to support struggling writers. Aligned with Veiga Simão et al. (2016) findings, most teachers reported implementing adaptations for struggling writers on a weekly basis or more often, with individual mentoring and extra sentence instruction more recurrently applied. Similar to findings from the U.S. context (Gilbert & Graham, 2010; Graham et al., 2003, 2006), instruction using computer technology was the least frequently reported adaptation for struggling writers in Chilean educational contexts.

## Implementing Instructional Adaptations: Teachers’ Preparation and Self-Efficacy

As proposed in the WWC model for writing (Graham, 2018), teachers’ competence and beliefs shape their teaching practices. Empirical studies investigating teaching practices in primary education, however, have indicated that primary school teachers find supporting struggling writers particularly challenging (de Abreu Malpique et al., 2023; Dockrell et al., 2016; Ralli et al., 2022). For example, Dockrell et al. (2016) found that 45% of teachers of younger (age 4–7) and older (8–11) students participating in their study in English primary schools thought that supporting struggling writers was problematic. In a similar study examining writing instruction in Greek primary schools (Grades 1–6), Ralli et al. (2022) also found that 41% of participating teachers thought that supporting struggling writers was difficult for them. In the Australian context, de Abreu Malpique et al. (2023) found that 47% of participating primary teachers (Grades 1–6) were not confident about their efficacy in adapting practices that support struggling writers.

Empirical research found preparation and self-efficacy for teaching writing to be the two most consistent predictors of writing instruction across educational contexts (Bañales et al., 2020; Graham, 2019), including in Australia (de Abreu Malpique et al., 2023). Very few studies, however, have examined the effects of teacher variables on instructional adaptations for writing, and some conflicting findings have been reported. National surveys in the United States (Graham et al., 2003, 2006, 2016) found that teachers’ self-efficacy to teach writing did not uniquely predict adaptations for struggling writers. However, Graham et al. (2022) found that both teachers’ preparation and efficacy to teach writing uniquely contributed to predicting Grades 4 to 5 teachers’ adaptations for struggling writers in Chile. Hence, further research examining the role of teachers’ preparation and beliefs in shaping instructional adaptations for writing is warranted. While findings from international surveys suggest that primary school teachers implement a variety of instructional adaptations for struggling writers, research findings are also indicative that the frequency of implementation and factors predicting the reported adaptations vary across countries. The present study examined primary grade teachers’ adaptations for struggling writers in Australian classrooms. This is, to our knowledge, the first study examining the adaptations that teachers typically make for struggling writers across Australian primary school settings.

## Research Questions and Hypotheses

The current study is part of a larger project investigating individual- and contextual-level factors impacting the writing performance of primary school students in Australia. In this study, we examined primary (Grades 1–6) teachers’ reported adaptations for struggling writers, addressing the following three research questions (RQs):

**RQ1:** How often do teachers make adaptations to teach writing for struggling writers in Australian primary classrooms (Grades 1–6)?
**RQ2:** Do teachers’ adaptations for struggling writers vary by grade?
**RQ3:** Do teachers’ perceived efficacy at teaching writing and their preparation to teach writing uniquely predict the reported instructional adaptations for writing?

Teachers were asked to report on the frequency with which they typically included specific adaptations for teaching writing to struggling writers in their classrooms (RQ1). The focus was placed on instructional adaptations that have been found to improve primary school students’ writing performance. For example, meta-analytic studies (e.g., Graham et al., 2012, 2017; Koster et al., 2015) show that struggling writers benefit from additional instruction in mastering basic skills, such as handwriting, spelling, and grammar, and in process writing skills, such as planning and revising texts. Hence, we asked teachers how often they provided extra teaching of the said writing skills and strategies. We further focused on adaptations to facilitate writing, such as pairing struggling writers with peers and providing additional minilessons to increase the learning of writing skills because there is evidence that these adaptations enhance writing performance (Danoff et al., 1993; Dowis & Schloss, 1992; Jasmine & Weiner, 2007). Considering prior research suggesting that primary teachers implement different adaptations to respond to the needs of struggling writers in their classrooms (Gilbert & Graham, 2010; Graham et al., 2003, 2006, 2022; Veiga Simão et al., 2016), we anticipated that most Australian primary teachers would report doing so on a weekly basis or more often. Previous studies found grade-level variability (RQ2) in teachers’ general reported practices for writing (de Abreu Malpique et al., 2023; Parr & Jesson, 2016). In a previous study investigating typical practices for writing instruction in Australian primary classrooms (de Abreu Malpique et al., 2023), Grade 1 teachers reported spending more time teaching spelling and handwriting than teachers in Grades 4 and 6, with results further suggesting that upper-primary teachers (Grades 4–6) placed a stronger emphasis on engaging students in planning and revising activities for text composing. Hence, we anticipated that the teaching of writing skills and strategies would vary across grades.

Regarding teachers’ preparation and perceived self-efficacy for teaching writing (RQ3), we expected that both variables would predict teachers’ instructional adaptations for writing because previous research found them to be the two most consistent predictors of writing instruction in primary classrooms in Australia (de Abreu Malpique et al., 2023). Teacher and classroom variables were found to account for variance in teachers’ reported practices for teaching writing in general (e.g., Bañales et al., 2020) and in teachers’ reported adaptations for struggling writers (Graham et al., 2003, 2006, 2022). Hence, we controlled for teaching experience because it was found to account for variance in teachers’ reported adaptations for struggling writers (Graham et al., 2003, 2006). We also controlled for variance due to gender because research has found associations among teaching practices, students’ performance, and teachers’ gender (Sabbe & Aelterman, 2007). Considering that teachers’ pedagogical practices for writing are influenced by academic and professional development opportunities (Veiga Simão et al., 2016), we also controlled for teachers’ educational level. Finally, we controlled for grade level because previous research has reported grade-level effects on instructional practices for writing in general (de Abreu Malpique et al., 2023) and on instructional adaptations for struggling writers (Graham et al., 2022), and time for writing practice as it was found to uniquely predict instructional adaptations for struggling writing in previous research with Grades 1 to 3 (Graham et al., 2003) and Grades 4 to 6 (Gilbert & Graham, 2010).

## Method

### Participants and Recruitment Procedures

In the Australian educational context, government and nongovernment schools (i.e., independent and catholic schools) are funded through a combination of Australian Government funding, state and territory government funding, and funding from fees and other parental or private contributions (Australian Government, Department of Education, n.d. a, b). Given time constraints related to seeking ethics approvals from government schools in Australia, which differ between states, independent schools were contacted in the first phase of the data collection process. A snowball sampling procedure was then used to recruit participants. The data collection process was conducted in three phases: (a) contacting all principals of the identified 1,449 primary independent schools compiled by the ACARA (n.d.). Principals were asked to share an information letter with their Grades 1 to 6 teachers inviting them to complete our online survey; (b) contacting relevant professional associations (e.g., Australian Primary Principals Association and Primary English Teaching Association Australia) to advertise the project and recruit participants from other primary school sectors (i.e., government and catholic); and (c) using online media as a means to advertise the project and recruit additional participants (i.e., Facebook and Twitter). Ethical restrictions prevented information being gathered about individual teacher’s school sectors to determine the sample’s representativeness per school sector (i.e., government, catholic, and independent).

Table 1 presents detailed information about participating teachers. Two hundred ninety-eight primary teachers (87.6% female) completed the survey scales developed for this study. The ideal sample size for the population of 152,820 primary school teachers in Australia (ACARA, n.d.) with a 5% margin of error and a 95% confidence level would be approximately 384. However, the actual sample size achieved for our study was 298. With this sample size, the calculated margin of error for a conservative estimate of population variance was approximately 5.67%, indicating that the sample estimates reported in this study offered a notable degree of precision. The distribution of survey participants across states was as follows: Western Australia (WA) constituted 26%, South Australia (SA) 16%, New South Wales (NSW) 15%, Tasmania (TA) 12%, Queensland (QLD) 10%, Australian Capital Territory (ACT) 9%, Northern Territory (NT) 7%, and Victoria (VIC) 5%. In comparison, the distribution of all primary school teachers in the broader population of Australia stood as follows: WA 10%, SA 7%, NSW 29%, TA 2%, QLD 21%, ACT 2%, NT 1%, and VIC 27%. While our sample cannot be judged as representative of the Australian population of primary teachers, it is important to highlight that some level of representation was reached for all states and territories.

**Table 1.** Characteristics of Australian Teacher Respondents to Survey.

Variable	*n*	%
Gender		
Female	261	87.6
Male	37	12.4
Other	—	—
State/territory where teaching		
WA	77	25.8
SA	49	16.4
NSW	45	15.1
TAS	35	11.7
QLD	30	10.1
ACT	27	8.7
NT	21	7.0
VIC	14	4.7
Highest education level		
Vocational	5	1.7
Bachelor	172	57.7
Graduate diploma	75	25.2
Masters	42	14.1
Doctorate	4	1.3
Years of teaching		
Mean	15.60 (*SD* = 8.87)	—
Median	15	—
Grade(s) currently taught		
1	41	13.8
2	54	18.1
3	45	15.1
4	60	20.1
5	51	17.1

### Survey Instrument

Participating teachers were asked to complete an online survey examining teaching practices in Australian primary classrooms, which included three sections. The first section asked teachers to provide demographic information, including gender, years spent teaching, and highest educational level. We further asked teachers to rate the quality of their college preparation to teach writing and the time they allocated for students’ writing practice in their classes. For Sections 2 and 3, we adapted items from Gilbert and Graham’s (2010) previous U.S. survey as it contained one scale assessing teachers’ perceived efficacy for teaching writing and another scale assessing adaptations for struggling writers, which were aligned with our research questions.

In Section 2, we asked teachers to rate their self-efficacy to teach writing. Five items assessed teachers’ perceived efficacy to teach writing and to adapt teaching practices to students’ individual needs. Teachers responded to each item using a 6-point Likert-type scale ranging from *strongly disagree* (score = 1) to *strongly agree* (score = 6), with a high score indicating stronger perceived efficacy. A factor analysis produced one single factor with an eigenvalue greater than 1.0 explaining 58% of variance, with all factors loading at 0.57 or higher and with coefficient alpha value of .81. The score for perceived efficacy at teaching writing was the average of the five items.

In Section 3, we asked teachers to indicate the frequency with which they implemented 16 different adaptations for struggling writers in their classrooms. Aligned with previous research in the field (Graham et al., 2016), teachers were not provided with a definition of the term *struggling writer* to allow them to interpret it in the context of their typical classrooms. In this section, adaptations included extra support and teaching beyond what teachers implemented with their typically developing writers. Teachers responded to this section using a 5-point Likert-type scale ranging from *never* (score = 1) to *daily* (score = 5). We tested the factorability of the 16 items and correlations for all list-wise combinations. The correlation matrix was appropriate for factor analysis, with a Kaiser–Meyer–Olkin measure of sampling adequacy of 0.82, which was well above the recommended value of 0.6. This exploratory factor analysis produced a three-factor solution, with eigenvalues suggesting that a two-factor solution was a better fit. A forced two-factor analysis with Varimax rotation to maximize factor dispersion revealed two items loading below .42, which were dropped (“extra encouragement” and “extra time for writing”). Subsequent analyses revealed 14 items explaining 63% of the overall variance. Eleven items loaded at .60 or higher on the first factor (eigenvalue = 6.62) accounting for 47.3% of variance. We named this factor as *Instructional Adaptations for Writing* as all items were focused on additional instruction in spelling, handwriting, capitalization and punctuation, grammar, sentence writing, text structure, planning and revising, as well as extra conferencing, minilessons, and reteaching of strategies and skills. Three items loaded at .77 or higher on the second factor (eigenvalue = 2.21) and accounted for 15.% of variance. We named this second factor as *Adaptations to Support Digital Writing* because two items directly focused on using computers for writing and one item on writing with the assistance of peers. We reasoned that this third item fit conceptually with the other items as computer-mediated collaborative writing was found to enhance primary students’ writing (Höysniemi et al., 2003; Storch, 2017). The score for each factor was the average of the retained items.

When completing the survey, teachers were asked to think about their typical practices for teaching writing and to select a class that they felt best represented their instructional practices for writing. Before data collection, the survey was field tested with four primary teachers who completed an initial version. Teachers were interviewed and asked to identify difficulties related to the survey’s administration and item interpretation, including time for completion, language issues, and items that could constrain participants’ responses. Subsequent changes were made on item clarity and wording.

## Results

As presented in Table 1, the responding teachers were mostly female (88%), generally held a bachelor’s degree or a graduate diploma (83%), and had on average about 16 years of teaching experience. Teachers from all states and territories are represented in the survey responses, with most respondents (57.3%) reporting they were teaching in Western Australia, South Australia, and New South Wales schools at the time of completing the survey. Table 2 presents descriptive statistics and bivariate correlations between predictors and control variables. Teachers reported allocating on average close to 3 hr a week for writing practices in their classes (*M* = 169.48 min, *SD* = 90.73, range = 15–450 min).

**Table 2.** Descriptive Statistics and Bivariate Correlations Between Predictors and Control Variables.

Variable	*M*	*SD*	1	2	3	4	5	6	7	8	9
1. Gender	1.88	0.33	1								
2. Years of teaching	15.60	8.87	.06	1							
3. Educational level	2.56	0.80	.03	.08	1						
4. Grade level taught	3.56	1.65	−.01	.24**	.26**	1					
5. Time for writing practice	169.48	90.73	.07	.19*	.21**	.40**	1				
6. Preservice preparation to teach writing	3.01	0.81	.01	.20**	.07	.14*	.41**	1			
7. Teacher self-efficacy	4.89	0.63	.10	.26**	.16**	.19**	.31**	.44**	1		
8. Instructional adaptations for writing	3.13	0.74	.10	.87	.08	.01	.12*	.02	.23**	1	
9. Adaptations to support digital writing	2.32	0.94	−.03	.63	.08	.16**	−.02	.07	.15**	.31**	1

* *p* < .05. **p < .01.

### Reported Preparation and Perceived Efficacy for Teaching Writing

When asked to rate the quality of preparation to teach writing they had received when completing their college degree, 42.6% of teachers indicated it was adequate, 27.9% very good, and 0.7% exceptional. Nearly 30% of teachers, however, reported that their preservice preparation to teach writing was poor (26.8%) or inadequate (2%). Teachers perceived self-efficacy for writing instruction was moderately positive (*M* = 4.88, *SD* = 0.64). Correlation analyses further showed that preparation was moderately correlated with perceived efficacy for teaching writing (*r* = .44, *p* < .001), indicating that teachers who felt better prepared at teaching writing also had higher levels of self-efficacy for writing instruction.

### Reported Instructional Adaptations for Writing

Table 3 presents frequency, means, and standard deviations for the 14 adaptations for struggling writers listed in the survey. Teachers reported only implementing three of the 14 adaptations more frequently (daily or at least once a week), namely, extra instruction on spelling (62.4%), sentence writing (61.5%), and capitalization and punctuation (56.7%). Teachers reported implementing seven of the 14 adaptations assessed on a monthly basis, namely, minilessons, additional instruction on grammar, handwriting, text structure, planning, revising/editing, and reteaching strategies and skills. The least frequently reportedly implemented adaptations for struggling writers were using technology to support writing (88.9%), using computers for writing (85.7%), and providing extra opportunities to write with peer assistance (83.8%).

**Table 3.** Frequency of Specific Adaptations for Struggling Writers.

Variable	Never (%)	At least once a year (%)	At least once a month (%)	At least once a week (%)	Daily (%)	*M* (*SD*)
Adaptations for writing						
Extra spelling instruction	3	5.7	28.9	46.3	16.1	3.67 (0.92)
Extra sentence instruction	3.7	5.4	29.5	48.7	12.8	3.61 (0.91)
Extra capitalization and punctuation instruction	1.3	9.1	32.9	43.6	13.1	3.58 (0.87)
Extra conferencing	8.1	21.5	26.2	34.2	10.1	3.17 (1.12)
Extra text structure instruction	5.7	12.4	47.3	30.2	4.4	3.15 (0.90)
Extra grammar instruction	11.1	12.8	39.3	29.9	7	3.09 (1.07)
Extra minilessons	4	18.1	51.7	23.8	2.3	3.02 (0.82)
Reteaching strategies and skills	5.4	22.5	55	10.7	6.4	2.90 (0.89)
Extra revising/editing instruction	10.1	26.5	38.3	19.1	6	2.85 (1.04)
Extra handwriting instruction	12.4	25.2	34.6	22.1	5.7	2.84 (1.08)
Extra planning instruction	17.8	26.2	31.9	19.1	5	2.67 (1.12)
Adaptations to support digital writing						
Peer assistance	23.8	31.5	28.5	12.8	3.4	2.40 (1.08)
Computer use	23.8	31.9	29.9	12.1	2.3	2.37 (1.05)
Technology to support writing	31.5	29.9	27.5	9.7	1.3	2.19 (1.03)

### Grade-Level Variance

For each of the 14 adaptations examined in the current study, a separate one-way analysis of variance (ANOVA) was computed to test grade-level variability and understand whether the teachers across primary grades (1–6) differed in how often they implemented an adaptation. Bonferroni correction (α of .05/14 analyses) was used to control for Type 1 errors, setting alpha at .003. Grade level was statistically related to the frequency of use of five adaptations for struggling writers, namely, extra instruction on text structure, *F*(5, 292) = 3.795, *p* = .002; revising/editing, *F*(5, 292) = 3.677, *p* = .003; spelling, *F*(5, 292) = 4.294, *p* = .001; handwriting, *F*(5, 292) = 5.846, *p* = .000; and computer use, *F*(5, 292) = 6.765, *p* = .000. Regarding extra instruction on text structure, first grade (*M* = 2.80, *SD* = 1.03) and third grade (*M* = 2.91, *SD* = 0.92) had statistically significant lower scores than fourth grade (*M* = 3.43, *SD* = 0.89). For extra revising/editing instruction, first (*M* = 1.80, *SD* = 0.93) and third grades (*M* = 2.00, *SD* = 0.93) had statistically significant lower scores than fourth grade (*M* = 2.30, *SD* = 1.14). For extra spelling instruction, first (*M* = 3.98, *SD* = 1.04) and second (*M* = 3.94, *SD* = 0.71) grades had statistically significant higher scores than third grade (*M* = 3.29, *SD* = 0.94). For extra handwriting instruction, first grade (*M* = 3.49, *SD* = 0.78) had statistically significant higher scores than second grade (*M* = 2.83, *SD* = 1.00), third grade (*M* = 2.38, *SD* = 1.07), and fifth grade (*M* = 2.55, *SD* = 1.04). Finally, for extra use of computer, first grade (*M* = 1.76, *SD* = 0.86) had statistically significant lower scores than fourth (*M* = 2.67, *SD* = 1.05), fifth (*M* = 2.61, *SD* = 0.96), and sixth grades (*M* = 2.72, *SD* = 1.23), while second grade (*M* = 2.15, *SD* = 0.92) had significant lower scores than sixth grade.

### Predicting Teacher Adaptations for Writing

Hierarchical multiple regression analyses (MRA) were conducted to determine whether teachers’ perceived efficacy for teaching writing and preparation to teach writing uniquely predicted the reported adaptations for struggling writers, namely, instructional adaptations and adaptations to support digital writing, accounting for variance associated with control variables, including teacher variables (gender, years spent teaching, and educational level) and classroom variables (grade level taught and amount of time for writing practice). Control variables were entered on Step 1 of the hierarchical MRA, with teachers’ perceived efficacy and preparation entered on Step 2, allowing us to determine whether teachers’ perceived efficacy and preparation made a unique and statistically significant contribution to predicting the reported adaptations beyond teacher and classroom variables.

### Predicting Instructional Adaptations for Writing

On Step 1 of the hierarchical MRA, control variables accounted for a nonsignificant 3.3% of the variance in general adaptations, *R*^2^ = .03, *F*(5, 291) = 2.01, *p* = .077. On Step 2, teachers’ perceived efficacy and preparation accounted for an additional 4.4% of the variance in general adaptations, ∆*R*^2^ = .04, ∆*F*(2, 289) = 6.97, *p* < .001. In combination, teachers’ perceived efficacy and preparation as well as teacher and class variables explained 7.8% of the variance in instructional adaptations for writing, *R*^2^ = .08, adjusted *R*^2^ = .06, *F*(7, 289) = 3.49, *p* < .001. Across all control and predictor variables, only teachers’ perceived efficacy for teaching writing made a unique and statistically significant contribution in predicting instructional adaptations (see Table 4 for unstandardized [*B*] and standardized [β] regression coefficients).

**Table 4.** Unstandardized (B) and Standardized (β) Regression Coefficients for Predicting General Adaptations.

Variable	*B* (95% CI)	β	*sr* ^2^
Self-efficacy	0.28 [0.13, 0.43]***	0.24	.04
Preparation	0.11 [−0.01, 0.23]	0.12	.01
Gender	0.15 [−0.10, 0.41]	0.07	.00
Years spent teaching	0.00 [−0.01, 0.01]	0.04	.00
Educational level	0.04 [−0.06, 0.15]	0.05	.00
Grade level taught	−0.04 [−0.09, 0.02]	−0.08	−.01
Time spent writing	0.00 [0.00, 0.00]	0.10	.01

*Note.* CI = confidence interval; *sr*^2^ *=* squared semi-partial correlation coefficient.

***p < .001.

### Predicting Adaptations to Support Digital Writing

On Step 1 of the hierarchical MRA, control variables accounted for a significant 4.1% of the variance in adaptations to support digital writing, *R*^2^ = .04, *F*(5, 291) = 2.49, *p* < .05. On Step 2, teachers’ perceived efficacy and preparation accounted for an additional 2.2% of the variance in adaptations to support digital writing, ∆*R*^2^ = .02, ∆*F*(2, 289) = 3.46, *p* < .05. In combination, teachers’ perceived efficacy and preparation as well as teacher and class variables explained 6.3% of the variance in adaptations to support digital writing, *R*^2^ = .06, adjusted *R*^2^ = .04, *F*(7, 289) = 2.80, *p* < .01. Across all control and predictor variables, only teachers’ perceived efficacy for teaching writing and class variables, namely, grade level taught and amount of time for writing practice, made a unique and statistically significant contribution in predicting adaptations for assisting digital writing (see Table 5 for unstandardized [*B*] and standardized [β] regression coefficients).

**Table 5.** Unstandardized (B) and Standardized (β) Regression Coefficients for Predicting Adaptations for Assisting Digital Writing.

Variable	*B* (95% CI)	β	*sr* ^2^
Self-efficacy	0.20 [ 0.01, 0.40]*	0.14	.01
Preparation	−0.07 [−0.22, 0.09]	−0.06	−.00
Gender	−0.10 [−0.42, 0.23]	−0.03	−.00
Years spent teaching	0.00 [−0.01, 0.01]	0.01	.00
Educational level	0.06 [−0.08, 0.20]	0.05	.00
Grade level taught	0.11 [ 0.03, 0.18]**	0.18	.03
Time spent writing	−0.00 [−0.00, 0.00]**	−0.17	−.02

*Note.* CI = confidence interval; *sr*^2^ *=* squared semi-partial correlation coefficient.

* *p* < .05. **p < .01.

## Discussion

Despite theoretical and empirical research arguing for the need to tailor educational practices to respond to students’ individual differences and needs, including in writing, there is a scarcity of studies investigating instructional adaptations that teachers typically make to support struggling writers in their classrooms (Graham, 2019). Indeed, most previous studies have been conducted in U.S. primary educational contexts, with only two studies carried out outside the U.S. context (Graham et al., 2022; Veiga Simão et al., 2016). The current study extends previous research by examining the variety and frequency of adaptations that teachers report they typically make to support struggling writers in Australian primary classrooms, as well as the role of teachers’ preparation and self-efficacy in predicting such reported instructional adaptations.

### Instructional Adaptations for Struggling Writers

Findings from the present study suggest that when making adaptations for struggling writers, primary teachers in Australia tend to place a particular focus on providing extra instruction on basic writing skills. When asked to report on the frequency with which they provided additional adaptations, the majority of respondents indicated that they provided extra instruction in spelling, capitalization and punctuation, and sentence construction at least once a week or more often, making these lower-level skills the three more commonly reported adaptations for struggling writers from the 14 adaptations assessed. Moreover, our findings suggest that junior primary teachers (Grades 1 and 3) placed less emphasis on providing additional text structure and revising/editing instruction than fourth-grade teachers, suggesting greater emphasis on basic skills in the first years of primary education. Our findings replicate previous studies developed in the United States (Gilbert & Graham, 2010; Graham et al., 2003, 2016) and Chile (Graham et al., 2022) reviewed here. The focus on mechanical aspects of writing may be associated with a number of factors, namely, a traditional emphasis on using text generation to assess mechanical and grammatical “correctness” (Connors, 1985, p. 2); the high prevalence of poor spelling, capitalization, punctuation, and grammatical errors evidenced by struggling writers (Grünke & Leonard-Zabel, 2015); and the positive impact that interventions that target these lower-level skills have on the writing outcomes of students with writing difficulties (Connelly & Dockrell, 2016). An intriguing finding emerging from our data is that, while teachers in junior years reported more support for handwriting than teachers in the senior primary years, overall, teachers did not report additional handwriting instruction as frequently as extra opportunities to teach punctuation, spelling, and grammar. This issue potentially speaks to a research-practice gap given substantive evidence that shows the critical role that handwriting automaticity plays on writing production and quality (Dockrell et al., 2019; Malpique et al., 2017, 2020). Consistent with previous surveys with nonstruggling writers (de Abreu Malpique et al., 2023), our current findings show that process writing skills, such as planning and revising texts, receive less emphasis than transcription and basic writing skills when supporting struggling writers. This is of concern as research has shown that teaching explicit strategies for planning and revising following a self-regulated strategy development approach positively impacts the writing performance of struggling writers (Graham & Harris, 2005). More substantial efforts are, therefore, required to expand teachers’ knowledge of evidence-based writing instruction for struggling writers that go beyond the teaching of basic skills.

On a more positive note, our findings do suggest that most teachers in our sample typically make a wide range of different adaptations for their struggling writers, with 11 of the 14 adaptations made by most teachers monthly or more often. These included extra minilessons and reteaching of strategies and skills, as well as additional instruction on grammar, handwriting, text structure, revising, and planning. Overall, findings from the current study are well aligned with previous studies showing that most primary teachers implement adaptations for struggling writers on a monthly basis or more often (Gilbert & Graham, 2010; Graham et al., 2016; Veiga Simao et al., 2016). In the Australian context, capacity for differentiated instruction is a professional standard that teachers as well as preservice teacher education programs must demonstrate to maintain accreditation (Australian Institute for Teaching and School Leadership [AITSL], n.d.). Given the increased diversity of students argued earlier in this article and the concomitant expectations for differentiation driven by bodies that regulate the teaching profession, it is not surprising to see primary teachers in Australia reporting a wide range of adaptations for struggling writers. While acknowledging that this is a positive finding, we argue that there are still significant gaps in teachers’ knowledge of evidence-based practices to support writing development.

The current study’s findings are also consistent with previous national surveys showing that primary teachers do not frequently use technological aids to support struggling writers in their classrooms (Gilbert & Graham, 2010; Graham et al., 2016, 2022). While our study results showed that Grades 4 to 6 teachers reportedly provided more additional use of computers for struggling writers than Grade 1 teachers, the highest average score for Grade 6 teachers was still one of the lowest among the 14 adaptations assessed in our study. Meta-analytic results from Morphy and Graham (2012) show that word processing contributes to enhanced motivation, writing quality, organization, length, and mechanical correctness among struggling writers (moderate to high effect sizes). Moreover, given empirically established connections between reading and writing (Graham, 2020), the use of technological aids such as e-readers can support struggling writers in observing “how good writing is organised and demonstrated in words, sentences and paragraphs” (Dunn, 2021, p. 3). Explicit instruction in the use of technological aids is, therefore, necessary for struggling writers not only to remove barriers to composing high quality texts, but also to prevent their existing literacy gaps being compounded with future struggles in their digital literacy. However, macro-level factors may make the integration of technological aids and word processing instruction in the learning and teaching of writing particularly challenging in Australian classrooms. Namely, findings from a recent survey examining Australian teachers’ use of digital technologies (K-12) (Zagami, 2022) suggest that laptop computers are more recurrently used in high schools, with a reported use of “bring your own device” (BYOD) programs in only 6% of lower primary schools. Moreover, as highlighted by Gulson et al. (2022), there is a “significant digital divide among Australian schools . . . as well as [issues related to] the skills and knowledge required to navigate technology” among school communities and teachers (p. 19). Hence, and given the emergence of digitalization in the last two decades, further attention must be placed on resourcing schools as well as on providing teachers with professional development opportunities to learn how to use technological aids to support struggling writers.

### The Role of Preparation and Self-Efficacy for Instructional Adaptations

Previous research examining predictors of primary-grade teachers’ instructional practices for writing has consistently reported that teachers’ perceived preparation and self-efficacy to teach writing uniquely predict general writing practices (Graham, 2019), including in Australia (de Abreu Malpique et al., 2023). In the current study, we examined the extent to which these two teacher variables explained variance in teachers’ adaptations for struggling writers after controlling for other teacher and classroom variables. We found that teachers’ self-efficacy for writing uniquely predicted both the instructional adaptations for writing and adaptations to support digital writing that we assessed. Research reports inconsistent findings regarding the role of self-efficacy in predicting teacher’s adaptations for struggling writers. As previously noted, only Graham et al. (2022) found a unique statistically significant contribution of teachers’ self-efficacy in predicting the use of adaptations for struggling writers in Chile. In a similar survey examining writing adaptations for SWDs, Troia and Graham (2017) found that teachers’ self-efficacy predicted the use of instructional supports for SWDs, but did not predict other subsets of adaptations, including technology aids. As proposed in the recent WWC model for writing acquisition and development (Graham, 2018), teaching practices are likely to be influenced by teachers’ beliefs about their own capabilities to teach writing. Considering this theoretical underpinning, the scarcity of studies examining the value of self-efficacy beliefs in predicting teacher adaptations, and inconsistent findings across studies and educational contexts, further research should focus on examining the unique contributions of this teacher variable in different contexts of instruction.

Our results further showed that, after controlling for teacher and classroom variables, teachers’ perceived preparation to teach writing did not predict the instructional adaptations for writing nor the adaptations to support digital writing here assessed. These findings are aligned with Troia and Graham’s (2017) reports showing a lack of contribution of teachers’ preparation in explaining technology aids to support SWDs. Our results, however, diverge from Troia and Graham’s (2017) U.S. study and Graham et al.’s (2022) Chilean study, where teachers’ preparation was found to predict instructional adaptations for writing. Our correlations analysis and subsequent regression analyses did not reveal relationships between teachers’ preparation and the adaptations for writing we assessed. As such, our results are better aligned with Veiga Simão et al.’s study (2016) showing no relationships between teachers’ preparation and adaptations for struggling writers in Portugal and Brazil. Our findings suggest that teachers’ efficacy may play a stronger role than teacher preparation in their enactment of practices that support struggling writers. In the Australian context, the absence of associations between perceptions of preparation and writing adaptations may speak, at least partly, to macro-level factors impacting writing instruction, namely, the nature of initial teacher education (ITE) programs in this area. According to a review of 27 primary and secondary ITE writing programs in New South Wales, “there is considerable variation across programs in the extent of content coverage, depth of treatment of relevant content and in what ITE students learn about effective teaching practice [in writing]” (New South Wales Education Standards Authority, 2018). Writing is a multidimensional and highly complex task, recruiting a wide range of cognitive, linguistic, and motor functions (Dockrell et al., 2019). Hence, teachers (preservice and in-service) need to be supported to acquire competence and confidence in implementing comprehensive evidence-based models of writing development and instruction while being equipped with efficient ways of assessing which writing skills (transcription and process skills) are being affected to provide targeted differentiated instruction. If one thing emerges clearly from our findings and previous research in writing adaptations for struggling writers is that there is much more to learn about what teachers are currently doing to support the wide range of writing abilities in their classrooms. This calls for greater diversity in methodological approaches, beyond self-reported surveys, to gain such an understanding.

### Limitations and Future Research

The present study has several limitations to be considered when interpreting findings and to inform future research. The first major limitation is the relatively small sample size of teachers from across Australian states and territories. A retrospective examination of our sample distribution highlighted certain disparities in representation, revealing both overrepresentation, as exemplified by WA, and underrepresentation, as observed in VIC, within our sample. Writing instruction in Australia is informed by national achievement benchmarks for writing (ACARA, 2021b), with different versions of the national curriculum implemented across states and territories to respond to contextual factors (Wall, 2017). Hence, our findings must be seen as a first attempt to gain insights regarding the variety and frequency of instructional adaptations for struggling writers provided in Australian primary classrooms. As variations between states and territories are likely to occur, future research investigating instructional adaptations for writing in specific states and territories, and reasons for their implementation, is warranted. Another limitation of the current study was the use of a self-report instrument, which does not assess real-time teaching practices. Following previous national surveys (e.g., Graham et al., 2003; Veiga Simão et al., 2016), we assumed that teachers would be aware of the nature of the teaching practices and able to relate these to the survey questions included in our online survey. Indeed, findings from prior teachers’ self-reported practices for writing (de Abreu Malpique et al., 2023; Graham et al., 2016; Malpique et al., 2017, 2020) have been corroborated by findings from observational studies (Coker et al., 2016, 2018; Guo et al., 2023; Puranik et al., 2014). While this is, to our knowledge, the first study investigating instructional adaptations for struggling writers across Australian primary classrooms, more research is needed to replicate and confirm our findings. Such research includes mixed-methods studies using interviews and observation protocols that capture the nature of teachers’ adaptations and the reasons explaining the use of specific instructional adaptations for struggling writers.

### Conclusion and Implications for Practice

Aligned with previous national surveys in the field, the findings from the current study suggest that primary teachers use a variety of instructional adaptations for struggling writers in Australian primary classrooms. While our results show that teachers implement these adaptations on a monthly basis or more often, they seem to be prioritizing the teaching of specific surface-level skills to support struggling. Empirical evidence shows the benefits of teaching handwriting (Barnett et al., 2020) and keyboard-based writing skills to support struggling writers (Morphy & Graham, 2012) and there is a strong body of research showing the importance of teaching planning and revising skills following a self-regulated strategy development approach for struggling writers (Kim et al., 2021). Meta-analytic findings (Guo, 2022) show that following a multicomponent approach to teaching writing is beneficial for struggling writers, so it would be important for teachers to provide additional instruction in both basic and process writing skills to respond to the needs of these students. Replicating findings from previous studies, our results were also indicative that teachers’ perceived efficacy for teaching writing uniquely predicted the instructional adaptations for writing that we assessed. Hence, attention must be placed on offering teachers high-quality education programs and professional development opportunities for writing instruction to support them in tailoring their teaching to students who find writing particularly challenging.

## References

[bibr1-00222194231211946] Australian Bureau of Statistics. (2019). Microdata: Disability, ageing and carers, Australia, 2018 (ABS Cat. No. 4430.0.30.002, AIHW analysis of Table Builder data).

[bibr2-00222194231211946] Australian Curriculum, Assessment and Reporting Authority. (n.d.). My school. https://www.myschool.edu.au

[bibr3-00222194231211946] Australian Curriculum, Assessment and Reporting Authority. (2020). The shape of the Australian Curriculum: Version 5. https://www.acara.edu.au/docs/default-source/curriculum/the_shape_of_the_australian_curriculum_version5_for-website.pdf?sfvrsn=2

[bibr4-00222194231211946] Australian Curriculum, Assessment and Reporting Authority. (2021a). Meeting the needs of student with a disability. https://www.australiancurriculum.edu.au/resources/student-diversity/meeting-the-needs-of-students-with-a-disability/

[bibr5-00222194231211946] Australian Curriculum, Assessment and Reporting Authority. (2021b). National assessment program: Literacy and numeracy. https://reports.acara.edu.au/NAP

[bibr6-00222194231211946] Australian Education Research Organisation. (2022). Writing development: What does a decade of NAPLAN data reveal? https://www.edresearch.edu.au/resources/writing-development-what-does-decade-naplan-data-reveal

[bibr7-00222194231211946] Australian Government, Department of Education. (n.d.-a). National report on schooling in Australia: Staff numbers. https://www.acara.edu.au/reporting/national-report-on-schooling-in-australia/staff-numbers

[bibr8-00222194231211946] Australian Government, Department of Education. (n.d.-b). Schooling: How schools are funded. https://www.education.gov.au/schooling/how-schools-are-funded

[bibr9-00222194231211946] Australian Institute for Teaching and School Leadership. (n.d.). Teacher standards. https://www.aitsl.edu.au/standards

[bibr10-00222194231211946] Bañales G. Ahumada S. Graham S. Puente A. Guajardo M. Muñoz I. (2020). Teaching writing in grades 4–6 in urban schools in Chile: A national survey. Reading and Writing: An Interdisciplinary Journal, 33, 2661–2696. 10.1007/s11145-020-10055-z

[bibr11-00222194231211946] Barnett A. L. Connelly V. Miller B. (2020). The interaction of reading, spelling, and handwriting difficulties with writing development. Journal of Learning Disabilities, 53(2), 92–95. 10.1177/0022219419894565 31847693

[bibr12-00222194231211946] Berninger V. W. Winn W. D. (2006). Implications of advancements in brain research and technology for writing development, writing instruction, and educational evolution. In MacArthur C. Graham S. Fitzgerald J. (Eds.), Handbook of writing research (pp. 96–114). Guilford Press.

[bibr13-00222194231211946] Coker D. L. Jr. Farley-Ripple E. Jackson A. F. Wen H. MacArthur C. A. Jennings A. S. (2016). Writing instruction in first grade: An observational study. Reading and Writing: An Interdisciplinary Journal, 29, 793–832. 10.1007/s11145-015-9596-6

[bibr14-00222194231211946] Coker D. L. Jr. Jennings A. S. Farley-Ripple E. MacArthur C. A. (2018). When the type of practice matters: The relationship between typical writing instruction, student practice, and writing achievement in first grade. Contemporary Educational Psychology, 54, 235–246. 10.1016/j.cedpsych.2018.06.013

[bibr15-00222194231211946] Connelly V. Dockrell J. (2016). Writing development and instruction for students with learning disabilities: Using diagnostic categories to study writing difficulties. In MacArthur C. A. Graham S. Fitzgerald J. (Eds.) Handbook of writing research (pp. 349–363). Guilford Publications.

[bibr16-00222194231211946] Connors R. J. (1985). Mechanical correctness as a focus in composition instruction. College Composition and Communication, 36(1), 61–72. 10.2307/357607

[bibr17-00222194231211946] Danoff B. Harris K. R. Graham S. (1993). Incorporating strategy instruction within the writing process in the regular classroom: Effects on the writing of students with and without learning disabilities. Journal of Reading Behavior, 25(3), 295–322. 10.1080/10862969009547819

[bibr18-00222194231211946] de Abreu Malpique A. Valcan D. Pino-Pasternak D. Ledger S . (2023). Teaching writing in primary education (grades 1–6) in Australia: A national survey. Reading and Writing: An Interdisciplinary Journal, 36, 119–145. 10.1007/s11145-022-10294-2 PMC9069425 35530430

[bibr19-00222194231211946] Dockrell J. E. Connelly V. Arfé B. (2019). Struggling writers in elementary school: Capturing drivers of performance. Learning & Instruction, 60, 75–85. 10.1016/j.learninstruc.2018.11.009

[bibr20-00222194231211946] Dockrell J. E. Marshall C. R. Wyse D. (2016). Teachers’ reported practices for teaching writing in England. Reading and Writing: An Interdisciplinary Journal, 29(3), 409–434. 10.1007/s11145-015-9605-9 PMC4761376 26941477

[bibr21-00222194231211946] Dowis C. L. Schloss P. (1992). The impact of mini-lessons on writing skills. Remedial and Special Education, 13(5), 34–42. 10.1177/074193259201300506

[bibr22-00222194231211946] Dunn M. (2021). The challenges of struggling writers: Strategies that can help. Education Sciences, 11(12), 795. 10.3390/educsci11120795

[bibr23-00222194231211946] Gilbert J. Graham S. (2010). Teaching writing to elementary students in grades 4 to 6: National survey. Elementary School Journal, 110(4), 494–518. 10.1086/651193

[bibr24-00222194231211946] Graham S. (2018). A revised writer (s)-within-community model of writing. Educational Psychologist, 53(4), 258–279. 10.1080/00461520.2018.1481406

[bibr25-00222194231211946] Graham S. (2019). Changing how writing is taught. Review of Research in Education, 43, 277–303. 10.3102/0091732x18821125

[bibr26-00222194231211946] Graham S. (2020). The sciences of reading and writing must become more fully integrated. Reading Research Quarterly, 55, S35–S44. 10.1002/rrq.332

[bibr27-00222194231211946] Graham S. Ahumada S. Bañales G. Puente A. Guajardo M. Muñoz I. (2022). Intermediate-grade teachers’ adaptations for weaker writers: A national survey in urban schools in Chile. Journal of Learning Disabilities, 55(2), 87–98. 10.1177/0022219420941795 32684066

[bibr28-00222194231211946] Graham S. Collins A. Rigby-Wills H. (2017). A meta-analysis examining the writing characteristics of students with learning disabilities and normally achieving peers. Exceptional Children, 83(2), 199–218. 10.1177/0014402916664070

[bibr29-00222194231211946] Graham S. Harris K. R. (2005). Improving the writing performance of young struggling writers: Theoretical and programmatic research from the center on accelerating student learning. The Journal of Special Education, 39(1), 19–33. 10.1177/00224669050390010301

[bibr30-00222194231211946] Graham S. Harris K. R. Bartlett B. J. Popadopoulou E. Santoro J. (2016). Acceptability of adaptations for struggling writers: A national survey with primary-grade teachers. Learning Disability Quarterly, 39(1), 5–16. 10.1177/0731948714554038

[bibr31-00222194231211946] Graham S. Harris K. R. Fink-Chorzempa B. MacArthur C. (2003). Primary grade teachers’ instructional adaptations for struggling writers: A national survey. Journal of Educational Psychology, 95(2), 279–292. https://psycnet.apa.org/doi/10.1037/0022-0663.95.2.279

[bibr32-00222194231211946] Graham S. McKeown D. Kiuhara S. Harris K. R. (2012). A meta-analysis of writing instruction for students in the elementary grades. Journal of Educational Psychology, 104(4), 879–896. 10.1037/a0029185

[bibr33-00222194231211946] Grünke M. Leonard-Zabel A. M. (2015). How to support struggling writers: What the research stipulates. International Journal of Special Education, 30(3), 137–150. https://files.eric.ed.gov/fulltext/EJ1095015.pdf

[bibr34-00222194231211946] Gulson K. Thompson G. Swist T. Kitto K. Rutkowski L. Rutkowski D. Hogan A. Zhang V. Knight S. (2022). Automated essay scoring in Australian schools: Key issues and recommendations. Education Innovations White Paper Series ISSN 2653-6749. Sydney Social Sciences and HumanitiesAdvanced Research Centre (SSSHARC), University of Sydney, Australia.

[bibr35-00222194231211946] Guo Y. (2022). Does transcription instruction make writing interventions more effective? A meta-analysis [Doctoral dissertation]. University of British Columbia.

[bibr36-00222194231211946] Guo Y. Puranik C. Xie Y. Dinnesen M. S. Breit A. (2023). An observational study of writing instruction and practice in kindergarten classrooms. Literacy Research and Instruction, 62(2), 180–202. 10.1080/19388071.2022.2153765

[bibr37-00222194231211946] Hayes J. R. (1996). A new framework for understanding cognition and affect in writing. In Levy C. M. Ransdell S. (Eds.), The science of writing: Theories, methods, individual differences, and applications (pp. 1–27). Erlbaum.

[bibr38-00222194231211946] Höysniemi J. Hämäläinen P. Turkki L. (2003). Using peer tutoring in evaluating the usability of a physically interactive computer game with children. Interacting with Computers, 15(2), 203–225. 10.1016/S0953-5438(03)00008-0

[bibr39-00222194231211946] Jasmine J. Weiner W. (2007). The effects of writing workshop on abilities of first grade students to become confident and independent writers. Early Childhood Education Journal, 35, 131–139. 10.1007/s10643-007-0186-3

[bibr40-00222194231211946] Kim Y. S. G. Yang D. Reyes M. Connor C. (2021). Writing instruction improves students’ writing skills differentially depending on focal instruction and children: A meta-analysis for primary grade students. Educational Research Review, 34, 100408. 10.1016/j.edurev.2021.100408 35991709 PMC9390887

[bibr41-00222194231211946] Koster M. P. Tribushinina E. De Jong P. Van den Bergh H. H. (2015). Teaching children to write: A meta-analysis of writing intervention research. Journal of Writing Research, 7(2), 299–324. 10.17239/jowr-2015.07.02.2

[bibr42-00222194231211946] López-Escribano C. Martín-Babarro J. Pérez-López R. (2022). Promoting handwriting fluency for preschool and elementary-age students: Meta-analysis and meta-synthesis of research from 2000 to 2020. Frontiers in Psychology, 13, Article 841573. 10.3389/fpsyg.2022.841573 PMC9204270 35719569

[bibr43-00222194231211946] Malpique A. A. Pino-Pasternak D. Roberto M. S. (2020). Writing and reading performance in Year 1 Australian classrooms: Associations with handwriting automaticity and writing instruction. Reading and Writing: An Interdisciplinary Journal, 33(3), 783–805. 10.1007/s11145-019-09994-z

[bibr44-00222194231211946] Malpique A. A. Pino-Pasternak D. Valcan D. (2017). Handwriting automaticity and writing instruction in Australian kindergarten: An exploratory study. Reading and Writing: An Interdisciplinary Journal, 30(8), 1789–1812. 10.1007/s11145-017-9753-1

[bibr45-00222194231211946] Morphy P. Graham S. (2012). Word processing programs and weaker writers/readers: A meta-analysis of research findings. Reading and Writing: An Interdisciplinary Journal, 25(3), 641–678. 10.1007/s11145-010-9292-5

[bibr46-00222194231211946] National Center for Educational Statistics. (2012). The Nation’s Report Card: Writing 2011 (NCES 2012–470). Institute of Education Sciences, U.S. Department of Education. https://nces.ed.gov/nationsreportcard/pdf/main2011/2012470.pdf

[bibr47-00222194231211946] New South Wales Education Standards Authority. (2018). Thematic review of writing preparation to teach writing: Report of the initial teacher education review. https://www.educationstandards.nsw.edu.au/wps/portal/nesa/about/who-we-are/research/research-reports/teaching-writing

[bibr48-00222194231211946] Organization for Economic Cooperation and Development. (2019). TALIS 2018 results (Volume I): Teachers and school leaders as lifelong learners. 10.1787/1d0bc92a-en

[bibr49-00222194231211946] Parr J. M. Jesson R. (2016). Mapping the landscape of writing instruction in New Zealand primary school classrooms. Reading and Writing: An Interdisciplinary Journal, 29(5), 981–1011. 10.1007/s11145-015-9589-5

[bibr50-00222194231211946] Price D. Slee R. (2021). An Australian Curriculum that includes diverse learners: The case of students with disability. Curriculum Perspectives, 41(1), 71–81. 10.1007/s41297-021-00134-8

[bibr51-00222194231211946] Puranik C. S. Al Otaiba S. Sidler J. F. Greulich L. (2014). Exploring the amount and type of writing instruction during language arts instruction in kindergarten classrooms. Reading and Writing: An Interdisciplinary Journal, 27, 213–236. 10.1007/s11145-013-9441-8 PMC3932498 24578591

[bibr52-00222194231211946] Ralli A. M. Dimakos I. C. Dockrell J. E. Papoulidi A. (2022). Teacher practices for teaching writing in Greek primary schools. Reading and Writing: An Interdisciplinary Journal, 35(7), 1599–1626. 10.1007/s11145-022-10258-6

[bibr53-00222194231211946] Sabbe E. Aelterman A. (2007). Gender in teaching: A literature review. Teachers and Teaching: Theory and Practice, 13(5), 521–538. 10.1080/13540600701561729

[bibr54-00222194231211946] Storch N. (2017). Peer corrective feedback in computer-mediated collaborative writing. In Nassaji H. Kartchava E. (Eds.), Corrective feedback in second language teaching and learning: Research, theory, applications, implications (pp. 66–79). Taylor & Francis.

[bibr55-00222194231211946] Thomas D. P. (2020). Rapid decline and gender disparities in the NAPLAN writing data. The Australian Educational Researcher, 47(5), 777–796. 10.1007/s13384-019-00366-8

[bibr56-00222194231211946] Troia G. Graham S. (2017). Use and acceptability of adaptations to classroom writing instruction and assessment practices for students with disabilities: A survey of grade 3-8 teachers. Learning Disabilities Research & Practice, 32(4), 257–269. 10.1111/ldrp.12135

[bibr57-00222194231211946] United Nations Educational, Scientific and Cultural Organization. (2019). UNESCO strategy for youth and adult literacy (2020-2025). https://unesdoc.unesco.org/ark:/48223/pf0000371411

[bibr58-00222194231211946] Veiga Simão A. M. Malpique A. A. Frison L. M. B. Marques A . (2016). Teaching writing to middle school students in Portugal and Brazil: An exploratory study. Reading and Writing: An Interdisciplinary Journal, 29(5), 955–979. 10.1007/s111

[bibr59-00222194231211946] Wall L. (2017). Institutional logics and curriculum decision making: Enacting the Australian curriculum English and NAPLAN literacy. The Australian Educational Researcher, 44(4), 391–407. 10.1007/s13384-017-0240-0

[bibr60-00222194231211946] Zagami J. (2022). Computer Education in Australian Schools: Enabling the next generation of IT professionals. Australian Computer Society. https://www.acs.org.au/insightsandpublications/reports-publications/ACS-Digital-Technologies-Education-Whitepaper_A04_FA_WEB.html

